# Feasibility of Tailoring Artificial Intelligence–Assisted Ambient Scribes for Intensive Care Unit Rounds: Algorithm Development and Validation

**DOI:** 10.2196/85015

**Published:** 2026-07-07

**Authors:** Ritchie Verma, Sandeep S Bains, Sai Harshith Reddy Muthani, Arun Arunachalam, Vishnu Mohan, Jeffrey A Gold

**Affiliations:** 1 Department of Medicine, Yale School of Medicine, 1450 Chapel St, New Haven, CT, 06511, United States, 1 (203) 789-3000; 2 Division of Informatics, Clinical Epidemiology, and Translational Data Science (DICE), Department of Medicine, Oregon Health and Science University, Portland, OR, United States; 3 Arizona State University, Tempe, AZ, United States; 4 Amazon, Seattle, WA, United States

**Keywords:** intensive care unit, clinical documentation, ambient scribe, artificial intelligence, large language model, prompt engineering

## Abstract

**Background:**

The increasing documentation burden on physicians is a significant contributor to burnout and decreases in care quality. Artificial intelligence (AI) has been proposed as a solution to reduce documentation burden in clinical care, but there are very limited data on its use in the inpatient and intensive care unit (ICU) environments.

**Objective:**

This pilot study aimed to explore the feasibility of using AI-assisted ambient scribes to capture interprofessional ICU rounds and synthesize a singular document to improve documentation efficiency and clinician satisfaction during ICU rounds. In this paper, we showcase our findings from customizing prompts for large language models (LLMs) to generate and evaluate daily progress notes from transcripts of simulated ICU cases.

**Methods:**

This project is divided into 2 phases. In the first phase, a randomly selected transcript of an audio recording of a simulated ICU rounds case was used to iteratively evaluate and improve the prompts for the LLMs. Multiple models (n=5) were used in phase 1, and the best-performing model (M1, based on the highest accuracy) was selected for the next phase. In the subsequent phase, 5 cases were selected and evaluated using the refined prompt and 2 models: M1 from phase 1 and M6, a technological upgrade of M1. Accuracy and error percentages were used as primary metrics. Additionally, error severity and usability were assessed using the Harm scale (adapted for potential harm risk) from the Agency for Healthcare Research and Quality and the 9-item Physician Documentation Quality Instrument, respectively.

**Results:**

Iterative improvements to the prompt increased accuracy and reduced errors during phase 1. In phase 2, M1 and M6 achieved accuracies of 69% and 80%, respectively (*P*=.04). Overall, errors of omission were most common (mean 15.5%, SEM 2.7%), followed by partial errors (mean 7.2%, SEM 0.92%) and then errors of commission (mean 2.6%, SEM 0.7%). The error severity of both models was low (*µ*=0.61 vs 0.53; *P*=.10), with most errors categorized as having potential for no harm to low harm. Both models performed well on the 9-item Physician Documentation Quality Instrument assessment, with the M6 model outperforming the M1 (35.8 vs 38.3; *P*=.06).

**Conclusions:**

Our findings demonstrate the feasibility of integrating AI-assisted scribes for ICU documentation. Both prompt improvements and technological advancements in LLMs are noted to be helpful. This study lays the groundwork for future research into AI applications in ICU settings, paving the way for broader improvements in health care documentation.

## Introduction

Physicians spend considerable time on documentation, a major risk factor for physician burnout [1]. This can lead to many clinicians leaving health care [2]. Furthermore, documentation burdens decrease patient care quality and increase medical errors [2]. Artificial intelligence (AI) has been proposed as a solution to address these health care challenges, including reducing the documentation burden [3,4]. Reducing clinical documentation should provide clinicians with more time to focus on patient care. This has the potential to improve provider satisfaction, patient safety, and quality of care while also saving substantial health care costs [4-6].

Recently, there has been a significant increase in the use of AI-assisted scribes in health care, especially in the outpatient setting and in primary care [7-10]. Many vendors have developed different versions of this product [11,12]. There are 2 primary components to the use of AI scribe technology [11]. First, the AI scribes capture ambient sound and generate a transcript, known as automated speech generation. The second component of AI scribes uses large language models (LLMs) to convert transcripts into AI-generated notes. Early results from outpatient implementations demonstrate reductions in documentation time, leading to greater efficiency, decreased physician workload, improved patient engagement, and increased physician satisfaction [7,9,10,13,14].

However, the use of this technology remains underexplored in inpatient and intensive care unit (ICU) settings. To our knowledge, no studies have examined the use of AI scribe technology in the ICU. ICU rounds are significantly different from comparable workflows in other clinical environments [15,16], especially those in the outpatient domain, where 1 clinician typically sees 1 patient in a private room, sometimes accompanied by one or more family members and/or friends. However, in the ICU setting, patient information is discussed during daily rounds [16]. The rounds are usually conducted in an open area outside the patient rooms within the ICUs. ICU rounds typically involve an interdisciplinary team with several members present, including an attending physician, trainees at different levels of graduate medical education, nurses, respiratory therapists, pharmacists, and potentially others, depending on the patient and the ICU staffing model [16]. Finally, in addition to the numerous members within each team, multiple teams frequently conduct rounds in the same clinical environment. Members of one or more teams may speak at the same time. Task and conversation interruptions are also quite common [17-19]. This makes the task of using AI-assisted ambient scribes much more challenging.

We aim to assess the feasibility of AI scribe technology in the ICU. In this pilot study, we assess the feasibility of AI-assisted ambient scribes for capturing interprofessional rounds and synthesizing a singular document. We focused on customizing the prompt for the LLM to generate daily progress notes from transcripts of simulated ICU rounds. The process involved prompt engineering to better align these notes with ICU settings and improve their quality. After optimizing the prompt, we performed early evaluations of the notes using the best-performing model during customization and its upgraded version released during the study.

## Methods

### Overview

**Figure 1.**
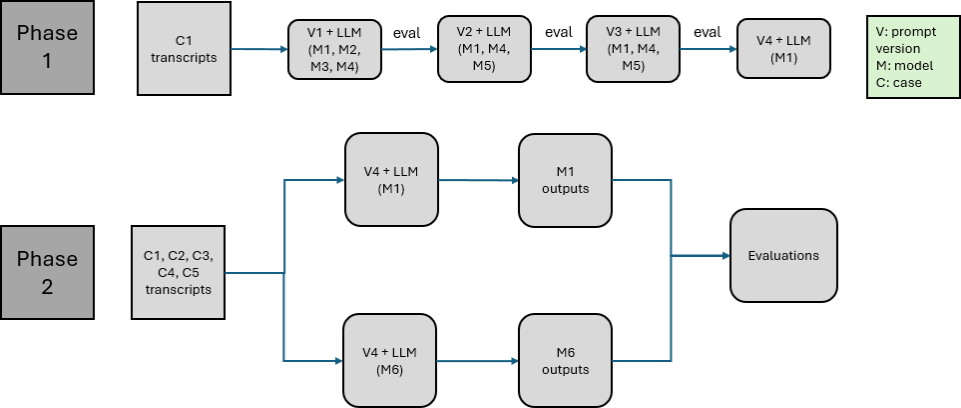
Overview of the 2 phases of the study. Phase 1 involved iterative prompt improvements from version 1 to version 4, using the output quality from case 1 and 5 large language models. Phase 2 involved evaluating outputs from 2 models across 5 cases using prompt version 4. C1, C2, C3, C4, C5: case numbers; eval: evaluation; LLM: large language model; M1, M2, M3, M4, M5, M6: model names; V1, V2, V3, V4: prompt versions.

To address the challenge of documentation burden in the ICU setting, we aimed to explore the use of AI-assisted ambient scribes. This project was divided into 2 phases. Phase 1 focused on customizing the prompt for the LLM component of the AI-assisted scribes for ICU rounds, while phase 2 involved evaluating the quality of the generated notes using a customized prompt. Figure 1 illustrates the process map of our project.

### Data

We used software-generated and manually reviewed transcripts of audio recordings from simulated rounds designed by domain experts. The underlying cases were derived from real-world examples. The simulations took place in a real ICU environment to ensure high fidelity. A detailed description of this process can be found in an earlier paper by Bordley et al [20] from our lab.

### Phase 1

#### Overview

In phase 1 of the project, 1 case (C1) was randomly selected to evaluate LLM prompt customization. This case was then run through 5 different models (M1, M2, M3, M4, M5; descriptions in Multimedia Appendix 1) to generate an ICU progress note. Only models (Claude and Llama family) available through the Health Insurance Portability and Accountability Act (HIPAA)–compliant Amazon Web Services Control Tower at Oregon Health & Science University were used in this study.

#### Evaluations

In this phase, evaluations were conducted by 2 physicians (RV, SSB) with backgrounds in internal medicine and critical care, serving as clinical subject-matter experts. The case (C1) was broken down into key clinical data elements using the ICU progress note structure as criteria. Daily progress notes are generally divided into different sections using the SOAP (Subjective, Objective, Assessment, and Plan) note structure [21]. Physicians then independently classified these data elements as accurate or erroneous. Errors were classified into 3 subtypes using a previously validated taxonomy [22,23]: error of omission (the element was present in the transcript but absent in the output note), error of commission (the element was absent in the transcript but present in the note), and partial errors (either parts of the element were inaccurate or omitted). The average of the 2 raters was used for final calculations. For quantitative evaluation, the percentages of errors (including subtypes) and accuracy were calculated for each output. For qualitative evaluations, consistent themes were assessed within the errors. The prompt underwent 3 iterative rounds of updates incorporating insights from these evaluations: V1→V2, V2→V3, V3→V4. Table 1 details the changes made to each prompt version, and Multimedia Appendix 1 provides a detailed description of each version of the prompt with Multimedia Appendix 2 provides the exact prompts. Only 1 model was selected to move to phase 2 based on its consistently highest accuracy and the lowest error rate.

**Table 1.** Prompt updates.

Prompt version	Update	Rationale
V1	Initial draft Introduced a hierarchical framework—main instructions detailing precise tasks, formatting guidelines, followed by a comprehensive medical record format with defined sections. Each section demands detailed extraction and organization from the ICU^a^ transcript for clarity, completeness, and actionable continuity of care documentation	The initial prompt was structured based on an ICU documentation template created by team members with domain expertise
V2	Assigned role—a prompt engineering method instructing the model to perform explicitly as a clinical documentation assistant. Modularly organized instructions—include an Instructions section guiding the model on medical record structure, tone, style, and content expectations, and a Special Instructions section that refines the model’s output by correcting specific behaviors, enforcing completeness, and ensuring inclusion of critical clinical details. Explicit direction for a thorough review of the transcript	Errors of omission were the biggest concern in the first iteration of the prompt involving both Subjective/24-hour events and Plan sections, so explicit language was added to the prompt to ensure completeness
V3	Introduced a new Orders & Medications subsection to capture and ensure inclusion of clinical details, such as new or discontinued treatments, that may not explicitly fit within other predefined sections of the medical record. Stronger compliance and review instructions—enhanced the Special Instructions section to ensure detailed content in the Assessment and Plan, requiring each subsection to progress from observation to action, with explicit documentation of current status, identified concerns, and clearly defined intervention plans	Errors of omission, specifically in the Plan section of the note, led us to introduce this new subsection to ensure that the detailed plan is captured in the transcript. In earlier versions, a few outputs already included this subsection, but we decided to make it mandatory
V4	Optimized for model-specific behavior: tailored the prompt specifically for the Claude model family by refining language to align with Claude strengths and response patterns, ensuring clearer comprehension and more accurate output generation. Added instructions to refine individual sections—such as relocated guidance for the Orders and Medications from Special Instructions into the <Medical Record Format> section, further classifying it into new and discontinued orders/medications	M1 was selected for subsequent evaluations, so the prompt was optimized to ensure consistency with the M1 family of models. Furthermore, the new subsection of Orders and Medications was broken down for clarity, as the data within this subsection included both instructions for new orders and discontinuation of old orders

a ICU: intensive care unit.

### Phase 2

#### Overview

Based on the results of phase 1, using the selection criteria described above, the team decided to proceed with the M1 model for phase 2 of the project. Of note, M5 also performed on par with the M1 model; however, this was discontinued during the study period, resulting in exclusion from phase 2. This phase involved evaluating the M1 model (Claude 3.5 Sonnet) using new simulated cases and prompt version V4. Additionally, a new model, Claude 3.7 Sonnet, became available as a direct upgrade from Claude 3.5 Sonnet. Five cases (C1 from phase 1 and 4 additional randomly selected cases—C2, C3, C4, and C5) were used in this phase of the study to assess whether the prompt generalizes well to other cases and to compare the performance of the new model (M6) with the model (M1) previously selected from phase 1.

#### Evaluations

Phase 2 evaluations were conducted by 3 physicians (RV, SSB, JAG) with clinical subject-matter expertise. JAG was included to leverage their expertise and provide additional objectivity, since JAG was not involved in developing the prompt, the list of clinical elements, or phase 1 of the evaluations. Similar to phase 1, RV and SSB broke down each case in phase 2 (C1-C5) into key clinical elements using the transcripts as ground truth. Hallucinations, that is, unsupported information noted in each output (M1 and M6 for each case), were added to this list of elements. For each case, there was only 1 list of elements (ie, the same number of total elements) to ensure comparability between the models. RV, SSB, and JAG then classified these elements into 4 categories (omission, commission, partial, correct), similar to phase 1. For quantitative evaluation, the percentages (with the denominator equal to the number of elements in each element list and equal weighting for each element) of errors (including subtypes) and accuracy were calculated for each output. To ensure consistency, interrater reliability was measured using Fleiss’ κ score for the preliminary rating. The kappa for the initial classification of errors and correct responses was 0.58 among all 3 raters. Discrepancies were resolved through collaborative review by all 3 raters until consensus was achieved. Errors in the transcripts were not graded as their limited number did not provide meaningful insights into the model’s behavior. Examples of transcript errors and model behavior are provided in Table S1 of Multimedia Appendix 1. We have also reported the error percentages for each SOAP category. As in phase 1, qualitative evaluations were performed to identify consistent themes within the errors.

Additionally, the error severity was evaluated separately by all 3 raters using the potential clinical harm of each error. We adapted the previously validated Agency for Healthcare Research and Quality (AHRQ) Harm Scale [23,24] for our study to assess the potential risk of harm associated with each error (Table S2 in Multimedia Appendix 1). Fleiss’ κ was calculated to evaluate interrater agreement. The κ for initial severity scoring was 0.91 among the 3 raters. Disagreement was defined as differences of 2 or more points between any 2 raters. Cases with disagreement (scoring differences of 2 or more) were further reconciled by all 3 raters. For all other cases, the average score of the 3 raters was used for analysis. The error severity score was categorized into 3 levels: no harm potential (AHRQ score=0), low harm potential (AHRQ score >0 and <2), and moderate-to-fatal harm potential (AHRQ score ≥2).

Finally, 2 raters (RV, SSB) rated the quality of the notes using the 9-item Physician Documentation Quality Instrument (PDQI-9) [25]. Additionally, note structure was also evaluated by comparing note length with errors and accuracy.

### Statistical Analysis

Using the percentages and themes of errors from phase 1 (described above), the prompt underwent 3 rounds of updates: V1→V4. Phase 2 evaluations involved comparing the accuracy and percentage of errors using prompt V4 and models (M1 and M6) across 5 separate cases (C1-C5), as described earlier. A Shapiro-Wilk test was used to check the normality of the data. Boxplots were created to check for outliers. A paired 2-tailed *t* test (data were normally distributed, and there were no outliers) was performed to compare the accuracy and errors (including subtypes) between M1 and M6. Bonferroni correction was applied where applicable for multiple comparisons. To compare the overall (combined M1 and M6) percentage of error subtypes, a Friedman test followed by Bonferroni correction with multiple comparisons was used, as the data were paired. Mean error severity scores were compared between M1 and M6 using a paired 2-tailed *t* test (normally distributed, no outliers). The chi-square test was used to compare the error severity categories between models M1 and M6. Spearman correlation analysis was conducted between accuracy and note length. A Wilcoxon signed-rank test was conducted to compare the average of PDQI-9 scores between M1 and M6 outputs. A *P* value of <.05 was considered statistically significant. All descriptive statistics are quoted as mean/percentages (SEM). All analyses were performed using Python (version 3.12.1). Figures were generated with the Python packages Matplotlib and Seaborn.

### Ethical Considerations

The study was deemed exempt by the institutional review board (7551) because only synthetic data were used to develop ICU rounds simulations. The synthetic data were derived from real-world cases covered under Institutional Review Board 9943.

## Results

### Phase 1

**Figure 2.**
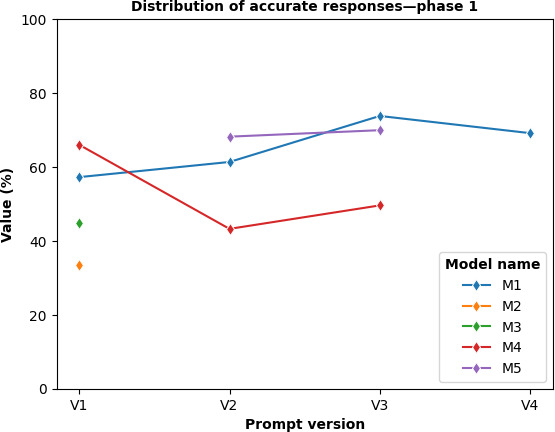
Percentages of accurate responses in phase 1. M1-M5 represent the models used in phase 1. V1-V4 represent the different prompt versions. Case C1 was used in phase 1.

Five separate models were evaluated in this phase of the study with different versions of the prompts (Table 2 and Figure 2). At each stage, team members used a combination of quantitative and qualitative assessments of the errors (Figures2  3 and Table 1) to decide which models would proceed. Most errors were related to omissions, and qualitative assessment revealed which sections and subsections of notes were most affected. These insights guided modifications to the prompts to address those errors. Models M2 and M3 performed poorly and were dropped early. M4 initially performed well but showed inconsistent results and a decline in performance after updates. Both M1 and M5 continued to perform well and responded positively to prompt updates at each stage. The team decided to proceed with M1 for the next phase of testing, as M5 was discontinued later in the study.

**Table 2.** Phase 1 (case number 1): accuracy and error scores for each model.

Model name and prompt version		Note length characters (words)	Omission (%)	Commission (%)	Partial (%)	Correct (%)
M1						
V1		2337 (364)	31.60	0.55	10.60	57.25
V2		2612 (420)	28.65	0	10.0	61.35
V3		3269 (515)	16.85	0	9.30	73.80
V4		3218 (519)	18.00	1.20	11.60	69.15
M2						
V1		2053 (303)	45.70	3.45	17.35	33.50
M3						
V1		2719 (444)	39.00	5.85	10.45	44.75
M4						
V1		4969 (751)	24.05	2.35	7.65	66.00
V2		2483 (383)	40.40	2.95	13.45	43.25
V3		3725 (588)	31.65	2.30	16.45	49.60
M5						
V2		3205 (513)	24.25	1.15	6.35	68.20
V3		3047 (510)	15.05	3.5	11.55	69.95

**Figure 3.**
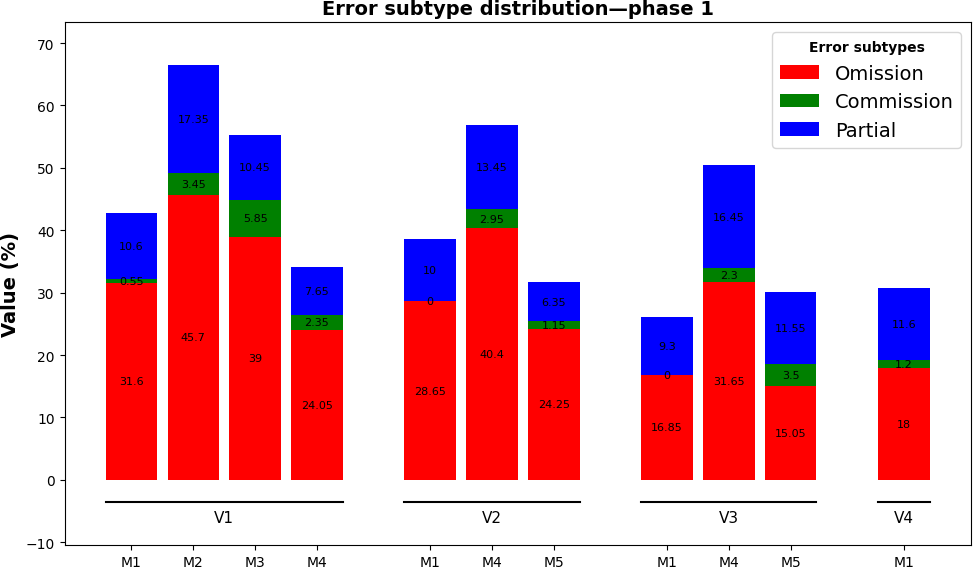
Breakdown of error subtypes in phase 1 by prompt version (V1-V4) and model name (M1-M5).

### Phase 2

**Figure 4.**
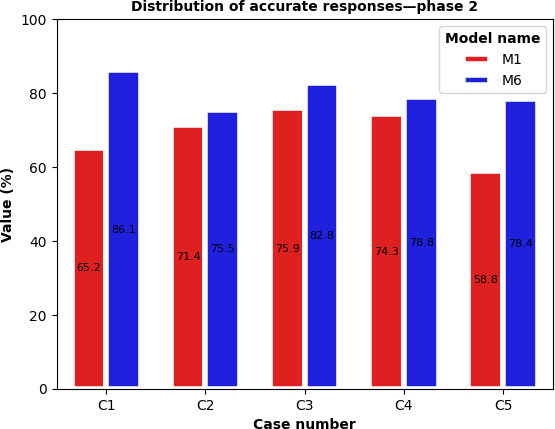
Percentages of accurate responses in phase 2. M1 and M6 represent models used in phase 2. C1-C5 represents 5 cases used in phase 2.

Based on phase 1 results, all phase 2 evaluations proceeded with version 4 of the prompt. Model M1 performed consistently across new cases, with an accuracy of 69.12% (95% CI 69.3%‐77.9%; Table 3 and Figure 4). In comparison, model M6, a direct upgrade of model 1 (Multimedia Appendix 1 for model description), performed better across all 5 cases, achieving an overall accuracy of 80.3% (95% CI 75.2%‐85.5%; *P*=.04).

**Table 3.** Phase 2 (prompt version 4): mean (SEM/SD) accuracy and error scores by each model.

Model name (n=5 cases)	Omission (%), mean (SEM/SD)	Commission (%), mean (SEM/SD)	Partial (%), mean (SEM/SD)	Accurate (%), mean (SEM/SD)
M1	21.60 (3.06/6.85)	1.30 (0.48/1.08)	7.90 (1.50/3.36)	69.12 (3.16/7.07)
M6	9.32 (2.12/4.75)	3.92 (0.97/2.18)	6.42 (1.13/2.53)	80.32 (1.85/4.15)

**Figure 5.**
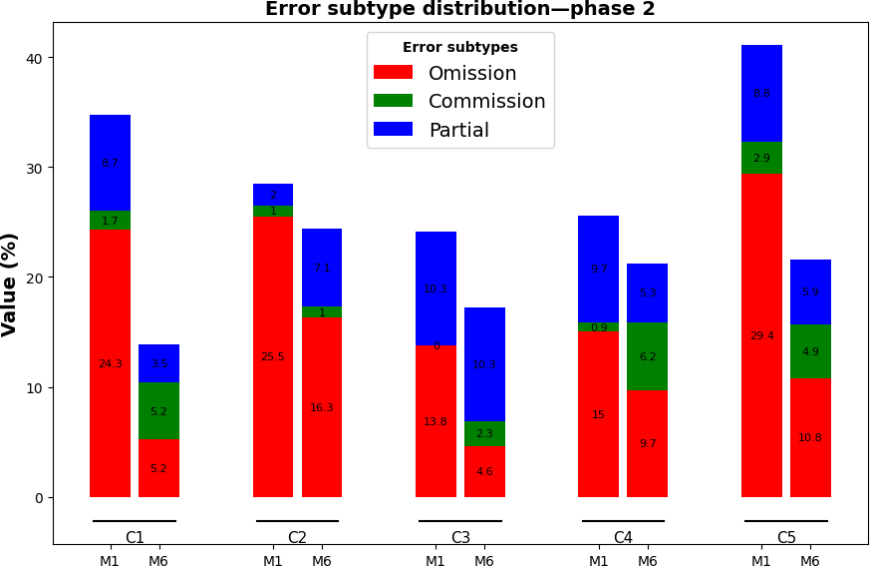
Breakdown of error subtypes in phase 2 by case number (C1-C5) and model name (M1 and M6).

Regarding error subtypes, combined (both M1 and M6) errors of omission were the most common (Figure 5) with an average of 15.5 (SEM 2.7%, 95% CI 9.4%‐21.6%), followed by partial errors at 7.2 (SEM 0.92%, 95% CI 5.1%‐9.3%), with errors of commission being the least common at 2.6 (SEM 0.7%, 95% CI 1.1%‐4.1%). Overall comparison of combined errors was statistically significant (*P*=.001), with subcomparison statistically significant only between omission and commission (adjusted *P*=.02). In terms of absolute counts, the errors of omission occurred at an average of 16 (SEM 2.85) errors per case, while partial errors occurred at 7.3 (SEM 0.89) errors per case, and errors of commission at 2.8 (SEM 0.76) errors per case. Comparison of error subtypes between the 2 models showed that only omission errors (21.60% vs 9.32%; adjusted *P* value =.03) were statistically significantly different between M1 and M6. On a deeper analysis of the errors, we compared which parts of the SOAP note structures contained the most errors. For M1, we observed that 5.08%, 2.75%, 1.95%, and 21.09% of the errors belonged to the Subjective, Objective, Assessment, and Plan categories, respectively. Similarly, for M6, these numbers were noted: 5.06%, 1.24%, 2.63%, and 10.76%, respectively. Further qualitative analysis of errors, with notable examples (Table 4), showed that they were related to historical data, vitals, lab data, imaging results, problems, differentials, and plans. M1 and M6 had similar errors of omission and partial errors, with the primary difference being quantitative: M6 had a lower proportion, as noted earlier. In contrast, errors of commission varied significantly by models, as noted in the examples in Table 4. Furthermore, most partial errors were omission-related.

**Table 4.** Qualitative results of error subtype analysis (phase 2).

Note category	Omission	Commission	Partial
History	Nurse’s concern for flu isolation (M1, M6) At home, the patient takes oxycodone (M1, M6) Recent intubation for pneumonia, now extubated (M1, M6)	None	Nurse concern for diminishing urine output and 10 liters positive (10 liters is omitted) (M1) Confirm with the patient if taking Hydrochlorothiazide at home with a plan to start later today after confirming (confirmation with the patient is omitted) (M1, M6)
Exam	Surgical site exam reported as “No report on surgical site” in the transcript (M1)	None	Mean arterial pressure is 79‐84, systolic 130 s with a blood pressure cuff (systolic reading off the cuff is omitted) (M1)
Lab	Glomerular filtration rate =54 (M1) Magnesium was 2 on 2/15 (admission) (M1)	None	Potassium—3.4, *was 3.1 more recently (recent comparison value is omitted*) (M1) Sodium—stable at 142 (Stable is omitted) (M1)
Imaging	“she has a CT chest from 2011, x-ray times two from this admission. no comment about the vertebral bodies.” (M1)	None	Chest X-Ray done while intubated on 2/22—Left lower lobe consolidation (while intubated is omitted) (M1)
Problem	“it’s become a thing that her pain medication is starting to interfere with the rest of her medical issues as well.” (M1) Streptococcal bacteremia (M1)	Blood counts are stable (M1) Transcript: “it’s become a thing that her pain medication is starting to interfere with the rest of her medical issues as well.” Output: “Pain is interfering with overall recovery and may be masking other symptoms.” (M6)	Blood Pressures (BP) are normal, hold off resuming BP meds (Blood pressures are normal is omitted) (M1) Creatinine is rising by 0.3 in the last 72 hours (last 72 hours is omitted) (M6)
Differential	New oversedation due to opioids (M1) Cause of patient’s mental status changes—medications (morphine, versed, oxycodone) (M1) Differentials include prerenal/volume overload related (M1)	“anemia noted, likely related to critical illness.” No discussion of causes of anemia in the transcript (M6) Differentials include infection and ICU^a^ stay for confusion. These causes are not discussed in the transcript (M6)	Differential Pulmonary Embolism given tachycardia/recent knee surgery (reasoning is omitted) (M1) Stop morphine due to worsening renal function and age (cause is omitted) (M1)
Plan	“Continue oxycodone 5‐10 q4h.” (M1, M6) “Repeat Basic Metabolic Panel at 2 PM.” (M1) Fluid balance positive, discontinue fluids (M1)	“No transfusion indicated at present, given stability.” The transcript has no discussion on transfusion. (M6) Code status: “will clarify with the patient.” The transcript does not include any discussion of code status. (M6) “Dispo: Continued ICU monitoring” (M1)	Start with X-ray lower back for imaging (output just says lower spine imaging—modality is omitted) (M1) Discontinue lacri-lube, mouthwash (Output says—various post-intubation meds) (M1)

a ICU: intensive care unit.

**Figure 6.**
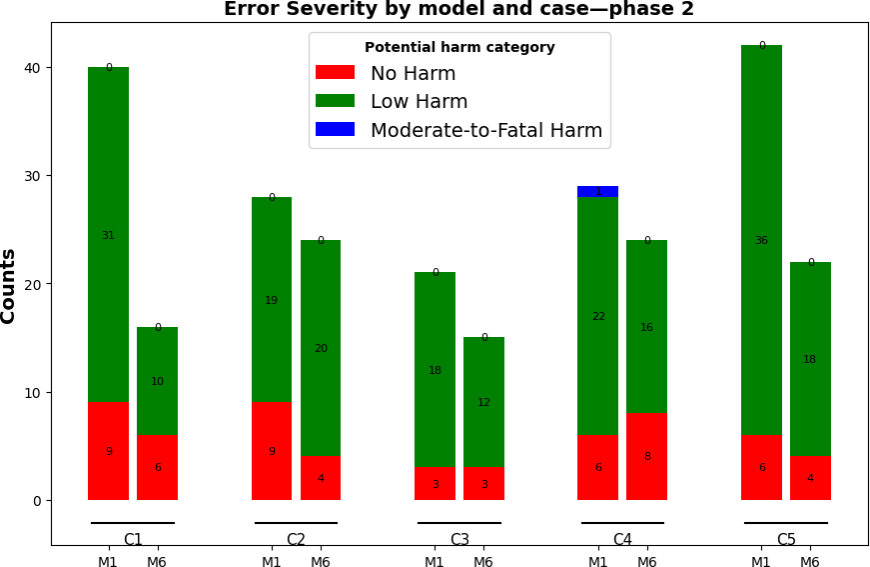
Breakdown of the error severity (harm potential) in phase 2 by case number (C1-C5) and model name (MA and M6). Error severity categories are defined as follows: (A) no harm: score=0; (B) low harm: score >0 and <2; (C) moderate-to-fatal harm: score ≥2.

The analysis of error severity showed that most errors (n) fell into the no harm or low harm categories (Figure 6). For the moderate-to-fatal harm category, M1 had errors at 0.2 (SEM 0.2) per case, whereas M6 had 0 (SEM 0). Furthermore, within the moderate-to-fatal category, no errors were classified as potentially severe or fatal. M1 had no harm errors at a rate of 6.6 (SEM 1.1) per case, compared to 5.0 (SEM 0.89) per case for M6, while low harm errors occurred at 25.2 (SEM 3.54) per case for M1 and 15.2 (SEM 1.85) per case for M6. The differences in severity between M1 and M6 were not statistically significant (*P*=.55). Table S2 in Multimedia Appendix 1 provides examples of each harm score category and their descriptions. Finally, the difference in average error severity scores between M1 and M6 (mean 0.61, SEM 0.03 vs mean 0.53, SEM 0.05; *P*=.10) was not statistically significant either.

In terms of note structure, the average note length for M1 and M6 outputs was 534.6 (SEM 35.7) and 1093.8 (SEM 57.5) words, respectively. Overall, a strong correlation was found between note length (*r*=+0.83; *P*=.003) and accuracy. Furthermore, note length had a very strong statistically significant negative correlation with errors of omission (*r*=−0.83; *P*=.003), and a strong positive correlation, albeit statistically not significant, with errors of commission (*r*=+0.62; *P*=.06). In contrast, there was no correlation with partial errors (*r*=−0.15; *P*=.66). Of note, when evaluated within models, the only significant correlation was between errors of commission and note length, within model M6 (*r*=+0.90; *P*=.04). Finally, in terms of overall readability and usefulness, M6 was better than M1 based on PDQI-9 scores, but the difference was not statistically significant (M1=35.8, SEM 0.78 vs M6=38.3, SEM 0.53; *P*=.06).

## Discussion

### Principal Findings

This study is the first of its kind to examine the feasibility of using AI-assisted ambient scribe technology in the ICU setting. Our findings show that the LLM component of the AI-assisted ambient scribes can be customized to capture interprofessional ICU rounds and produce a singular document aligned with ICU workflows. While the current literature is limited on the use of AI scribes in the ICU, our results suggest that the use of customized AI-assisted scribes in the ICU setting can generate similar outputs compared to outpatient AI-assisted scribe apps [26-28]. Our results demonstrate that prompt engineering plays a crucial role in improving note quality and customizing them for specific settings and formats. However, the variations observed between different LLMs using the same prompt indicate that technological optimization must address both areas. This aligns with other studies showing high variability in LLM models’ performance with identical prompts [29-31]. Our study highlights the need to further investigate the use of AI-assisted scribes in ICU settings and the potential benefits of conducting additional research to validate our findings in a larger, more diverse cohort and to evaluate the accuracy of automated speech recognition components in scribes and their correlation with note errors.

Existing documentation practices are also prone to errors. Data omission is particularly common, with 100% of ICU patient presentations containing at least one omission in 1 study [32]. In at least 1 study, when physician trainees prepared prerounding notes and verbal presentations, 22.9% of the data were missing from written artifacts and 42.4% from the oral presentations [32]. Another study shows laboratory data communication during ICU rounds was similarly problematic, with 96% of patient presentations including at least 1 laboratory misrepresentation and 38.9% of all audited laboratory data being inaccurately communicated [33]. Most of these errors were omissions rather than incorrect values, and only 7.8% were detected by the rounding team [33]. Our results show the viability of AI-scribe technology for accurately reflecting what is being discussed. However, as noted in these prior studies, interprofessional rounds in ICU settings are far from perfect, underscoring the need for standardization. In our study, we observed that most errors occurred in the Plan section, likely reflecting greater ambiguity in assessment and planning. However, the errors were in the objective or lab data in these prior studies. This further emphasizes the need to standardize verbal presentations; otherwise, we would just be compounding errors. Conversely, AI scribes should create notes that are free of copy-and-paste information, thereby reducing errors related to these mechanisms in the electronic health records [34]. In studies outside ICU settings, prior research has reported error rates ranging from 7% to 21% in clinical documentation, whether derived from dictation software (office notes, discharge summaries, and procedure notes) or patient-reported data (ambulatory notes) [35,36].

Upon closer examination of errors in AI-assisted scribe documentation output, we found that most originated from omissions. Therefore, our main focus in prompt engineering was to reduce missing elements in the generated note. One method to accomplish this was to add a gold-standard template to the prompt. The Plan section was divided into organ-based subsections, which helped identify issues related to specific areas, such as psychiatric problems including delirium and sedation, that were not always captured under the neuro subsection in earlier versions of the prompt. However, by explicitly including subsections for most organ systems in the note template, we observed that models sometimes generated plans for endocrine or hematology-oncology sections even when these were not discussed in the transcript. Although the generated content was not always incorrect or clinically significant, as shown in our qualitative results, for example, “anemia noted” when hemoglobin numbers are in the anemia range, this approach appeared to carry a risk of errors of commission, such as hallucinations or fabrications. Some errors could have a greater clinical impact, including statements such as “cause of anemia” or discussions of “transfusions.” This indicates a trade-off when using a more detailed template in the prompt to better capture data elements. Of course, this trade-off could be eliminated by using strategies to optimize the prompt to reduce errors of commission. This remains an area to be explored in future work.

To enhance completeness and reduce errors of omission, additional prompt engineering procedures included adding “New Orders,” “Discontinued Orders,” and a “Medications” subsection at the end of the note. This resulted in significantly improved performance. Interestingly, the transcripts also included a discussion at the end where the team listed the new and discontinued orders and medications for the day. This presents an opportunity for clinicians to adjust workflows to bring rounds closer to the note templates, thereby further improving transcript and note accuracy. However, future studies should examine the use of the ambient scribe in real-world ICU settings to ensure it does not impose additional tasks or cognitive burdens on the teams. This is especially important, given that the current safeguard against errors in AI scribe notes is manual review by clinicians, which adds an additional burden on clinicians and could lead to automation complacency and/or bias [37-42].

Besides prompt engineering, we found that upgrading LLMs also greatly enhanced performance. Once the prompt was set, using M6 (the larger model) yielded significantly better performance than the earlier version, M1. Part of this difference could be explained by M6’s ability to generate longer notes. We observed a strong overall correlation between note length and accuracy (and errors of omission); however, this correlation is primarily driven by between-model differences rather than a continuous within-model trend. Interestingly, there was a strong positive correlation between note length and overall errors of commission (primarily within model M6), although their impact on accuracy (and total error rate) was not large, as errors of commission constituted a much lower percentage of total errors. This shows a small trade-off between errors of omission and errors of commission depending on the model’s thoroughness or the length of the note. Although longer notes (model-dependent) appear to yield lower error rates, this could affect workflows, further burdening clinicians who would have to manually review longer notes. Ironically, as noted in another study [34], copy-and-pasted information increases the length of the notes, and AI-generated notes, in theory, should not contain copied-and-pasted information. Thus, generating longer notes by AI to reduce omissions might not have as much impact on workflows as anticipated and presents another opportunity for future research. This is evident in the perceived usefulness of these outputs in PDQI-9 scores, where the differences between model outputs are not statistically significant; in fact, the trend favors M6.

Another consideration when using ambient scribes in the ICU is privacy and confidentiality [41,42]. When assessing the feasibility of ambient listening devices in the ICU using the clinical rounds’ structure, which is generally an open environment (as opposed to office-based visits), we need to be mindful that these devices will capture speech from multiple clinicians, families, and ancillary staff. This has implications for consent procedures [41]. For example, would everyone in the ICU have to consent to AI scribes? What about people entering the ICU after rounds have started? Each state’s wiretapping laws may further complicate these regulatory issues [43]. Other regulatory considerations for the use of AI scribes include HIPAA compliance and data security [42], such as where these conversations are stored and for how long. Implementing institutional data governance policies, recording practice protocols, and keeping up with evolving guidance for AI-enabled tools is essential [44].

While the absolute number of individual errors was significant for both models, the severity of these errors was mostly low, categorized as no harm to low harm potential. Additionally, we observed that the error severity scores showed little difference between the 2 models. These results are encouraging, though it is too early to draw definitive conclusions given the small sample size and limited variation in clinical problems in our dataset.

Interestingly, in a few instances, LLMs seemed to extract the correct information from contextual clues, even when errors occurred in the transcripts. This is likely a probabilistic inference based on LLM training data. This could be a strength in ICU or inpatient settings, where open environments and ambient noise can affect transcript quality. However, the autonomous correction of clinical data without flagging it for a human being should be recognized as a potential safety risk. Incorporating safeguards, such as uncertainty signaling or explicit error flagging, could help reduce the cognitive burden of revisions needed after AI scribes generate a note. Since our data lacked sufficient errors in the transcripts to thoroughly analyze model behavior, this remains an area for future research.

### Limitations

There are some limitations to our study. First, we did not investigate whether automated speech recognition software impacts transcript quality in ICU settings. While this is important, it was out of scope for this initial pilot to assess the feasibility of customizing the prompt for the ICU-specific setting. We plan to address this in our future work on this project. Second, the small sample size and the use of synthetic cases lacking significant clinical variation might overestimate the accuracy and would limit the generalizability of our findings. We also kept case C1 (used for prompt tuning in the first phase) as a baseline in the second phase for comparison; this could have somewhat inflated the quantitative results, as the prompt was optimized specifically for case 1. Since our synthetic cases were developed from real-world ICU rounds, they partially captured the nonlinear reasoning and diagnostic uncertainty expected in the ICU setting. Additionally, audio simulations were developed using multiple speakers and a simultaneous multiple-team rounding structure, capturing acoustic noise. However, as stated earlier, the goal of this study was to explore the feasibility of our approach in ICU settings. This is early work, and a continuation of our project will build on this. As the inferential statistics are underpowered, our findings should be interpreted as strictly preliminary and hypothesis-generating rather than definitive evidence of performance across ICU populations. Third, we did not perform a head-to-head comparison with standard error rates in ICU notes. The 80% (approximately) accuracy should not be interpreted as evidence of superiority or inferiority relative to current clinical documentation practices. It is a reflection of agreement with the expert-defined content in a controlled environment. The goal of this paper was to assess the minimum safety level before we test this technology in a real-world environment. Fourth, we had only moderate agreement (κ=0.58), reflecting subjectivity in defining errors within the ICU notes. We addressed this by manually reviewing each disagreement together and revising the classification based on the combined consensus, thereby reducing the impact on measurements. This also reflects the inherent complexity of ICU documentation rather than just subjectivity among reviewers. Clinicians often differ in their judgments of the notes’ relevance and completeness. These reliability metrics should be interpreted as approximate indicators rather than definitive measures, suggesting the need to improve them in the future as well. Fifth, the evaluators were not blinded, which could have added confirmation bias, especially in subjective metrics such as partial errors (despite reviewing disagreements together) and PDQI-9 scores. Sixth, we used only models (Llama and Claude) available on the HIPAA-compliant Amazon Web Services Control Tower at Oregon Health & Science University. Since we empirically used Claude in phase 2, our results should not be generalized to all other LLMs. We noticed a trend toward larger language models performing better (M6 vs M1); however, it remains unclear whether this holds for other model classes. Future work is needed to evaluate prompt transferability to other LLMs. Seventh, our study did not evaluate whether M6’s superior performance was due to inherent improvements in reasoning or simply because its outputs were twice as long as those of M1. There is an apparent trend toward a trade-off between the conciseness and completeness of these AI-generated clinical notes. Future evaluations incorporating normalization strategies, such as accuracy per unit length of the note or trials of different prompts urging smaller models to write longer notes, could be helpful.

Our future goals build upon this work in three key areas: (1) evaluating AI scribes in diverse ICU cases and clinical conditions; (2) assessing the accuracy and quality of speech recognition, transcripts, and notes; and (3) studying the real-world use of ambient AI scribes to understand their impact on workflows and any potential unintended consequences.

### Conclusions

In conclusion, we examined the use of AI scribes in the ICU setting. By combining prompt engineering with multiple LLMs, we successfully generated ICU-specific notes with fairly high accuracy. The overall quality and usefulness received high ratings from team members with domain expertise. Analyzing the different types of errors offered valuable insights into areas where efforts could be directed to enhance the quality of these notes. Since AI scribes have seldom been used in ICU environments, our study offers an important starting point and valuable insights for further exploration of this application.

## Supplementary material

10.2196/85015 Multimedia Appendix 1 Model behavior related to transcript errors, error severity, description of models used in phase 1, description of models used in phase 2, and prompt version details.

10.2196/85015 Multimedia Appendix 2 Exact prompts for versions 1-4.

## Abbreviations

AHRQ: Agency for Healthcare Research and Quality
AI: artificial intelligence
HIPAA: Health Insurance Portability and Accountability Act
ICU: intensive care unit
LLM: large language model
PDQI-9: 9-item Physician Documentation Quality Instrument
SOAP: Subjective, Objective, Assessment, and Plan
