# Supplementary material for: Feasibility of Tailoring Artificial Intelligence–Assisted Ambient Scribes for Intensive Care Unit Rounds: Algorithm Development and Validation
Source: JMIR Med Inform. 2026 Jul 7;14:e85015. doi: 10.2196/85015 (PMC13340570; doi:10.2196/85015)
Supplement: Multimedia Appendix 1 [file medinform-v14-e85015-s001.docx]

**Multimedia Appendix 1**

Contents

[Table S1: Examples of errors in transcripts and models’ behavior in those situations 1](#_Toc221090331)

[Table S2. The Agency for Healthcare Research and Quality Harm Scale adapted for potential harm risk, along with examples 2](#_Toc221090332)

[Description of LLMs used in Phase 1 3](#_Toc221090333)

[Description of the new LLM used in phase 2 5](#_Toc221090334)

[Prompt Versions Details 6](#_Toc221090335)

## Table S1: Examples of errors in transcripts and models’ behavior in those situations

| Examples of transcription errors and models’ behavior in those situations |
| --- |
| 1. Potassium value in transcript was mis-transcribed as “450.0”. M1 and M6 both replaced it with “4.5” in the note. |
| 1. Bicarb value was mis-transcribed as “Bicarb looks good.  Increasing, actually, from 0.6 to 31 over the last several days.”. M6 replaced it with “increasing from 26 to 31 over several days,” whereas M1 used the values from the transcript, “Improved from 0.6 to 31”. |
| 1. RASS score was mis-transcribed as “VAS score”. Both models created an output with a “VAS score”. |
| 1. Hematocrit is mis-transcribed as “Her H&H, hemoglobin is 9.4, stable from 9.3 yesterday, creatinine of 27.3.”. Both models omitted the hematocrit in the output and mentioned only hemoglobin numbers. |

## Table S2. The Agency for Healthcare Research and Quality Harm Scale adapted for potential harm risk, along with examples

| Score | Description | Example – Omission | Example – Commission |
| --- | --- | --- | --- |
| 0 | No or negligible potential for harm. | Failure to capture when a patient was extubated (how many days ago, etc.) | Documented “No plan to transfuse” (transfusion plan not mentioned in transcript) |
| 1 | Mild harm potential. Bodily or psychological injury resulting in minimal symptoms or loss of function, or injury limited to additional treatment, monitoring, and/or increased length of stay. | Failure to capture Tylenol dosages or failing to completely capture the dosages of opioids that are continued at the same dose. | The cause of anemia is documented but not discussed in the transcript. |
| 2 | Moderate harm potential. Bodily or psychological injury adversely affecting functional ability or quality of life, but not at the level of severe harm. | Failure to capture the cause of confusion, such as pain medications/opioids | Captured Full code whereas code status not mentioned in the transcript |
| 3 | Potential for severe harm. Bodily or psychological injury (including pain or disfigurement) that interferes significantly with functional ability or quality of life. | None | None |
| 4 | Potential for harm resulting in death. | None | None |

## Description of LLMs used in Phase 1

M1 - Claude 3.5 Sonnet V2

Claude 3.5 Sonnet is an advanced AI language model developed by Anthropic, introduced in June 2024 with multimodal capabilities for text and visual data. It features a substantial context window of 200,000 tokens, enabling it to process extensive documents and maintain context over lengthy interactions. It excels in reasoning, coding, and automation, making it a versatile tool for professional and academic applications.

M2 - Claude 3.5 Haiku

Claude 3.5 Haiku, developed by Anthropic and released in October 2024, is a high-speed AI language model optimized for real-time applications. It features a 200,000-token context window, allowing it to process extensive documents and maintain context over lengthy interactions. The model excels in rapid data extraction, automated labeling, and real-time content moderation, making it ideal for tasks requiring quick response times, such as interactive chatbots and coding assistance.

M3 - Llama 3.1 70B

Llama 3.1 70B is a state-of-the-art large language model developed by Meta AI, released in July 2024. This model boasts 70 billion parameters and supports a context window of up to 128,000 tokens. Trained on approximately 15 trillion tokens from publicly available sources, Llama 3.1 70B demonstrates advanced capabilities in natural language understanding and generation. It is optimized for multilingual dialogue use cases, supporting eight languages. The model is available under an open-source license, facilitating research and commercial applications.

M4 - Llama 3.1 405B

Llama 3.1 405B is a state-of-the-art large language model developed by Meta AI, introduced in July 2024. With 405 billion parameters, it stands as one of the largest openly available foundation models to date. The model supports a context window of up to 128,000 tokens. Trained on approximately 15 trillion tokens from publicly available sources, Llama 3.1 405B demonstrates advanced capabilities in natural language understanding and generation. It is optimized for multilingual dialogue use cases, supporting multiple languages. The model is available under an open-source license, facilitating research and commercial applications.

M5 - Claude 3 Opus

Claude 3 Opus, introduced by Anthropic in March 2024, is the most advanced model in the Claude 3 series, designed for complex reasoning tasks. It features a 200,000-token context window, enabling it to process extensive documents and maintain context over lengthy interactions. Claude 3 Opus excels in advanced reasoning, mathematics, coding, and multilingual communication, demonstrating near-human comprehension and fluency. It also possesses strong vision capabilities, allowing it to process various visual formats, including photos, charts, and technical diagrams. This model is particularly suitable for tasks requiring high accuracy and deep understanding across diverse domains.

## Description of the new LLM used in phase 2

M6 - Claude 3.7 Sonnet

Claude 3.7 Sonnet, released by Anthropic in February 2025, is an advanced multimodal AI language model that builds upon the capabilities of Claude 3.5 Sonnet. It introduces a novel "hybrid reasoning" architecture. This flexibility enables the model to adapt its problem-solving approach based on the complexity of the task at hand. Claude 3.7 Sonnet features an expanded 250,000-token context window, enabling comprehensive analysis of extremely large documents and sustained, coherent interactions.

Additionally, it supports generating outputs of up to 128,000 tokens per response, facilitating detailed, nuanced, and context-rich replies suitable for extensive reports, in-depth analyses, and comprehensive summaries. Designed for professional and academic settings, it delivers enhanced accuracy, deeper context awareness, and improved responsiveness, making it exceptionally effective for applications demanding precision and extended reasoning capabilities.

## Prompt Versions Details

Prompt engineering involved three rounds of updates (V1-->V2, V2-->V3, V3-->V4), as described below. These changes were made iteratively based on the output from each version of the prompt using LLM models.

**V1:**

This prompt is structured using a hierarchical framework. It begins with general instructions that define tone, style, and format, followed by special instructions reinforcing completeness and detail, and concludes with section-specific guidance detailing expected content for each part of the medical record.

It employs explicit instruction-based prompting to ensure clarity, structured output enforcement through standardized section headers, and precise expectation setting by defining medical parameters, data types, and professional terminology. Additionally, it incorporates self-verification mechanisms to prevent missing information.

Tested outputs across four models: M1, M2, M3, and M4.

**V2:**

Role Priming & Task Clarity: Version 2 introduces a defined role ("clinical documentation assistant") and a clear task statement, improving context awareness and goal alignment for structured information extraction.
Instructions are now modularly organized using <Instructions> and <Special Instructions>, making them more digestible and hierarchical for better compliance. Stronger Emphasis on Completeness & Detail – Special instructions now explicitly direct a thorough review of the transcript and encourage more detailed documentation, especially in the Assessment and Plan section.

Narrowed focus to M1 and M4, with M5 inducted to evaluate test outcomes in this phase.

**V3:**

Special instructions now specify including orders and medications at the end of the record to ensure that treatments not fitting specific categories are clearly documented.

Tested by adding examples under each section of the medical record format to evaluate their impact on output accuracy but reverted due to negligible improvement.

Refined the prompt to optimize outcomes for Models M1 and M5.

Stronger compliance and review instructions, explicit directive to thoroughly verify and confirm adherence to all guidelines, ensuring the mandatory inclusion of all sections. Increased detail and logical structuring, emphasis on writing each section with sufficient depth for continuity of care.

**V4:**

Tested incorporating the HealthScribe-generated note alongside the transcript but reverted due to no performance improvement and the introduction of new errors.

Generated and evaluated outputs across various ICU round audio recordings.

Optimized to minimize errors from the previous iteration and refined the prompt to align with the behaviors of Model M1.

The section organization is refined for improved consistency. The 'Orders/Medications' instruction has been promoted from a special guideline to a dedicated section at the end of the medical record template, specifying the detailed inclusion of both new and discontinued orders to improve treatment tracking.
